# Yellow Fever

**Published:** 1879-04

**Authors:** 


YELLOW FEVER. By THOS. O. SUMMERS, M.D., Professor of Anatomy and Histology in the University of Nashville and Vanderbilt University. Copyrighted. Wheeler & Bros., publishers, Nashville, Tenn. 1879.

In this the etiology, pathology, clinical history, treatment and prophylaxis of this terrible disease are given at some length. The author asserts, and to our minds reasonably and correctly, that yellow fever is non-contagious; that it is indigenous, and that its *avant courier* is malarial developments. Could the rational, correct ideas portrayed in this little volume be impressed upon the skeptic minds of a large body who are straining themselves to inaugurate a rigid and universal quarantine, the realization of the fact would flash upon them that "we have already too long hugged the delusive phantom of quarantine, which is as inhuman as it is unscientific and impracticable."

